# The Bacterial Toxin CNF1 Induces Activation and Maturation of Human Monocyte-Derived Dendritic Cells

**DOI:** 10.3390/ijms19051408

**Published:** 2018-05-08

**Authors:** Laura Gall-Mas, Alessia Fabbri, Martin R. J. Namini, Michael Givskov, Carla Fiorentini, Thorbjørn Krejsgaard

**Affiliations:** 1 Department of Immunology and Microbiology, University of Copenhagen, Nørre Alle 14, 2200 Copenhagen, Denmark; lgmas@sund.ku.dk (L.G.M.); mnamini@sund.ku.dk (M.R.J.N.); 2 Italian Center for Global Health, Istituto Superiore di Sanitá; Viale Regina Elena 299, 00161 Rome, Italy; alessia.fabbri@iss.it (A.F.); carla.fiorentini@iss.it (C.F.); 3 Costerton Biofilm Center, Department of Immunology and Microbiology, University of Copenhagen, Nørre Alle 14, 2200 Copenhagen, Denmark; mgivskov@sund.ku.dk

**Keywords:** CNF1, bacterial toxins, dendritic cells, moDCs, Rho GTPases, cytokine expression, T-cell activation, inflammation, anti-virulence immunity, effector-triggered immunity

## Abstract

Cytotoxic necrotizing factor 1 (CNF1) is a bacterial protein toxin primarily expressed by pathogenic *Escherichia coli* strains, causing extraintestinal infections. The toxin is believed to enhance the invasiveness of *E. coli* by modulating the activity of Rho GTPases in host cells, but it has interestingly also been shown to promote inflammation, stimulate host immunity and function as a potent immunoadjuvant. The mechanisms underlying the immunostimulatory properties of CNF1 are, however, poorly characterized, and little is known about the direct effects of the toxin on immune cells. Here, we show that CNF1 induces expression of maturation markers on human immature monocyte-derived dendritic cells (moDCs) without compromising cell viability. Consistent with the phenotypic maturation, CNF1 further triggered secretion of proinflammatory cytokines and increased the capacity of moDCs to stimulate proliferation of allogenic naïve CD4+ T cells. A catalytically inactive form of the toxin did not induce moDC maturation, indicating that the enzymatic activity of CNF1 triggers immature moDCs to undergo phenotypic and functional maturation. As the maturation of dendritic cells plays a central role in initiating inflammation and activating the adaptive immune response, the present findings shed new light on the immunostimulatory properties of CNF1 and may explain why the toxin functions as an immunoadjuvant.

## 1. Introduction

Cytotoxic necrotizing factor 1 (CNF1) is a bacterial protein toxin produced by certain strains of pathogenic *Escherichia coli*. *cnf1*-positive *E. coli* have been isolated from children with diarrhea, but are most frequently associated with extraintestinal conditions, such as urinary tract infections, bacteremia and neonatal meningitis [1]. *E. coli* strains harboring the gene for CNF1 have also been isolated from skin and soft tissue infections, suggesting that the toxin may function as a virulence factor in diverse host niches [2]. After bacterial secretion, CNF1 enters host cells by receptor-mediated endocytosis [3]. Two cellular receptors have so far been shown to mediate CNF1 internalization; the 37/67 kDa laminin receptor and the Lutheran adhesion glycoprotein/basal cell adhesion molecule (Lu/BCAM) [4,5,6,7]. Following endocytosis, endosomal acidification and cleavage of CNF1 leads to translocation of the active part of the toxin into the cytoplasm where it deamidates a specific glutamine residue in Rho GTPase proteins. This modification impairs the intrinsic GTP hydrolyzing activity of Rho GTPases and thereby locks them in their active state. The Rho GTPases control a wide range of cellular processes but are best recognized for their central role in regulating the dynamics and organization of the actin cytoskeleton. Accordingly, CNF1 induces rearrangements of the actin cytoskeleton in target cells, leading to morphological and functional changes. These rearrangements can facilitate bacterial internalization into host cells, and substantial evidence supports that CNF1 contributes to the invasiveness of pathogenic *E. coli* by manipulating the epithelial and endothelial barriers [3,8,9].

Infection studies have further reported that CNF1 has the capacity to promote inflammation in vivo, indicating that the toxin also possesses immunostimulatory properties [10,11,12,13]. In line with this conclusion, CNF1 has been shown to be a potent immunoadjuvant that can augment antigen-specific adaptive immune responses and host protection [14,15,16]. A catalytically inactive variant of CNF1, where cysteine 866 has been mutated into a serine residue (CNF1-C866S), is devoid of immunoadjuvant properties, suggesting that the immunostimulatory capacity of the toxin is linked to its enzymatic activity [14,15]. Boyer and co-workers accordingly demonstrated that CNF1-mediated activation of the Rho GTPase Rac2 elicited an inflammatory response in *Drosophila melanogaster* that promoted eradication of pathogenic *E. coli* [17]. In addition, Diabate et al. found that *cnf1*-positive *E. coli* were cleared faster from the blood of bacteremic mice than isogenic *cnf1*-negative *E. coli* or *E. coli* expressing CNF1-C866S [18]. The rapid clearance of *cnf1*-positive *E. coli* was associated with improved survival of the mice and enhanced levels of cytokines, chemokines and Gr1-positive immune cells (e.g., granulocytes and inflammatory monocytes) in the blood. Antibody-mediated depletion of Gr1-positive cells was sufficient to block the eradication of *E. coli* during bacteremia and prevent the CNF1-induced bacterial clearance. CNF1 was further shown to potentiate lipopolysaccharide (LPS)-triggered expression of cytokines and chemokines from monocytes in vitro. These findings suggest a scenario in which CNF1 enhances the expression of inflammatory factors from activated monocytes leading to increased mobilization of Gr1-positive cells and thereby more effective clearance of bacteria in the bloodstream [18,19]. Accumulating data thus indicate that CNF1 activity stimulates a protective inflammatory response in host organisms. However, the mechanisms that contribute to CNF1-induced tissue inflammation remain unclear, and little is known about the direct effects of the toxin on different immune cell subsets.

Dendritic cells (DCs) are innate immune cells that play a pivotal role in stimulating the inflammatory response against intruding pathogens and activating adaptive immunity. They exist in two distinct functional and phenotypic states, known as immature and mature. Immature DCs are widely distributed throughout the body, where they function as key sentinels of infection in tissues and at mucosal surfaces. In the immature state, DCs are generally tolerogenic, but upon sensing invading microbes, they are activated to undergo a maturation process where they upregulate expression of major histocompatibility complex (MHC) and co-stimulatory molecules to become potent antigen-presenting cells that are highly efficient at activating naïve T cells. Furthermore, activated DCs secrete inflammatory cytokines that contribute to the activation and recruitment of various immune cells and regulate naïve T cell differentiation. Immature DCs can sense invading microbes via a battery of pattern recognition receptors (PRRs) that recognize specific microbe-associated molecular patterns (MAMPs) [20,21,22]. MAMPs are, however, present in both pathogenic and non-pathogenic microbes, implying that DCs employ additional mechanisms to discriminate between them. Although this phenomenon remains poorly characterized in DCs, a growing body of evidence supports that host cells may assess the pathogenic potential of bacteria by sensing virulence factor- and toxin-induced manipulation of fundamental cellular processes [23]. Given the reported immunostimulatory properties of CNF1, we therefore investigated the capacity of the toxin to induce activation and maturation of monocyte-derived DCs (moDCs).

## 2. Results

**Figure 1:**
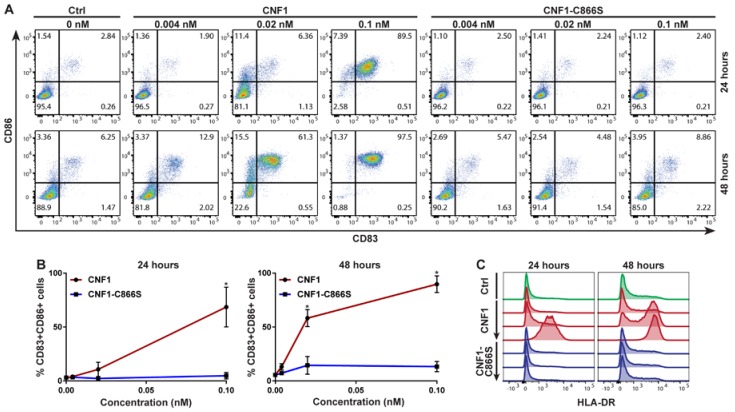
Cytotoxic necrotizing factor 1 (CNF1), but not CNF1-C866S, induces phenotypic maturation of immature monocyte-derived dendritic cells (moDCs). (**A**–**C**) Immature moDCs were cultured for 24 and 48 h without toxin (Ctrl) or with different concentrations of CNF1 or CNF1-C866S before the expression of phenotypic maturation markers was measured by flow cytometry. (**A**) Representative pseudocolor dot plot showing surface expression of CD83 and CD86. (**B**) Graphs summarizing the mean percentages of CD83+CD86+ cells from 3 independent experiments, each using moDCs derived from different donors (*n* = 3). Error bars represent SEM and * denotes a significant difference (*p* < 0.05) compared to immature moDCs cultured without toxin using a two-way ANOVA with Sidak’s multiple comparisons test. (**C**) Histogram plots showing the surface expression of HLA-DR on moDCs cultured without toxin (green), with CNF1 (red) or with CNF1-C866S (blue). Arrows indicate increasing concentrations (0.004, 0.02 and 0.1 nM) of CNF1 and CNF1-C866S. The plots are representative of 3 independent experiments each using moDCs derived from different donors (*n* = 3).

To address whether CNF1 induces DC maturation, we initially cultured human immature moDCs with different concentrations of wild-type or catalytically inactive CNF1 (CNF1-C866S) for 24 or 48 h and analyzed the surface expression of the two classic DC maturation markers CD83 and CD86. Importantly, CNF1 triggered a dose- and time-dependent increase in the percentage of CD83 and CD86 double-positive cells when compared to immature moDCs cultured without toxin (Figure 1A,B). Furthermore, CNF1 induced increased surface expression of HLA-DR MHC class II molecules in a similar dose- and time-dependent fashion, collectively demonstrating that the toxin elicits phenotypic maturation of immature moDCs (Figure 1C). The moDCs exhibited a remarkable sensitivity to CNF1; relative to immature moDCs cultured without toxin, as little as 20 pM CNF1 induced a strong and significant increase in the percentage of CD83 and CD86 double-positive cells within 48 h of culture (Figure 1A,B). In contrast, CNF1-C866S had no significant impact on the expression of the analyzed maturation markers, indicating that CNF1 triggers moDC maturation via its catalytic activity (Figure 1A–C).

**Figure 2:**
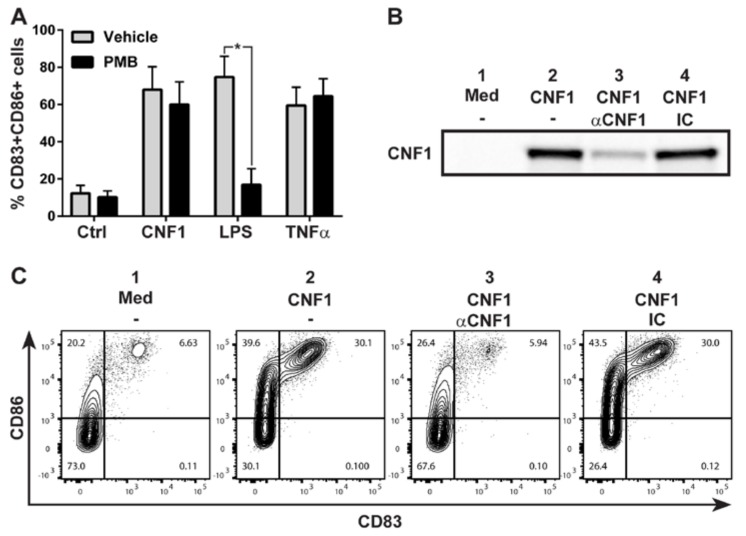
CNF1-induced moDC maturation is not driven by endotoxin or other putative contaminants in the toxin preparation. (**A**) Culture media alone (Ctrl) or culture media containing 0.1 nM CNF1, 50 ng/mL lipopolysaccharide (LPS) or 50 ng/mL tumor necrosis factor alpha (TNFα) was pre-incubated for 30 min at room temperature with 5 µg/mL Polymyxin B (PMB) or vehicle (endotoxin-free water). Then, immature moDCs were added to each media sample and cultured for 24 h before the expression of CD83 and CD86 was determined by flow cytometry. Shown is the mean percentage of CD83+CD86+ moDCs from 4 different donors (*n* = 4) analyzed in 3 independent experiments. Error bars represent SEM and * denotes a significant difference (*p* < 0.05) between vehicle- and PMB-treated cells using a paired Student’s *t*-test. (**B**) CNF1 diluted in moDC medium was incubated with (2) uncoated Protein A agarose beads or (3) Protein A agarose beads that had been pre-coated with a monoclonal anti-CNF1 (αCNF1) antibody or (4) an isotype control (IC) antibody. As a negative control, (1) moDC medium without CNF1 was in parallel incubated with uncoated Protein A agarose beads. After removal of the beads, the level of CNF1 in the supernatants was analyzed by western blotting. (**C**) Immature moDCs were cultured for 24 h with the supernatants (1–4) obtained in (**B**) and the expression of CD83 and CD86 analyzed by flow cytometry.

Products purified from Gram-negative bacteria can contain substantial levels of endotoxin (LPS), which is a potent inducer of moDC maturation. It was therefore essential to confirm that the observed maturation of moDCs was mediated specifically by CNF1 and not due to LPS contamination of the toxin preparation. To this end, we employed the polypeptide antibiotic Polymyxin B (PMB), which binds to and neutralizes LPS with very high affinity [24]. As judged by the frequency of CD83 and CD86 double-positive cells, PMB completely blocked LPS-induced maturation of moDCs, whereas it had no significant effect on cytokine-induced (TNFα) or, importantly, CNF1-induced moDC maturation (Figure 2A). While this result provided evidence that the CNF1-induced maturation of moDCs was not due to the presence of LPS in the CNF1 toxin preparation, it did not exclude that it could be caused by other putative bacterial contaminants. We therefore diluted the CNF1 toxin preparation in culture media and incubated aliquots with uncoated agarose beads, agarose beads coated with a specific antibody against CNF1 or agarose beads coated with an isotype control antibody. As a negative control, culture media without CNF1 was incubated with uncoated agarose beads. The agarose beads were subsequently removed by centrifugation and, as anticipated, agarose beads coated with CNF1-specific antibody depleted CNF1 from the toxin preparation when compared to aliquots incubated with uncoated agarose beads or agarose beads coated with isotype control antibody (Figure 2B). Depletion of CNF1 from the toxin preparation strikingly abrogated its capacity to induce CD83 and CD86 expression on immature moDCs whereas toxin preparations that had been incubated with uncoated agarose beads or agarose beads coated with isotype control antibody retained their ability to trigger moDC maturation (Figure 2C). Altogether, these results strongly support that the observed maturation of moDCs was mediated specifically by CNF1 and not by LPS or other putative contaminants in the toxin preparation. This conclusion was further corroborated by our prior data, showing that CNF1, but not CNF1-C866S, triggered moDCs maturation even though the toxins were purified using the same procedure (Figure 1).

**Figure 3:**
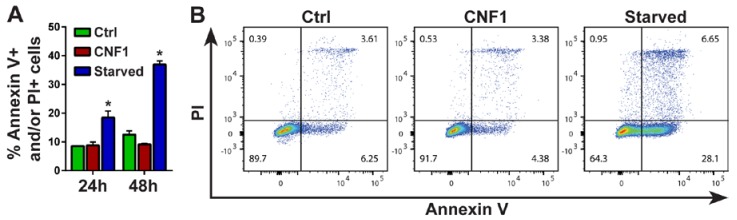
CNF1 does not induce apoptosis or cell death in moDCs. (**A**,**B**) Immature moDCs were cultured for 24 and 48 h without toxin (Ctrl) or with 0.1 nM CNF1 before the percentages of Annexin V- and propidium iodide (PI)-positive cells were determined by flow cytometry. moDCs cultured in medium without fetal bovine serum and cytokines (Starved) were included in the analysis as a positive control for induction of apoptotic cell death. (**A**) Histogram summarizing the mean percentages of Annexin V+ and/or PI+ cells from 3 independent experiments, each using moDCs derived from different donors (*n* = 3). Error bars represent SEM and * denotes a significant difference (*p* < 0.05) compared to Ctrl moDCs at the same timepoint using a two-tailed paired Student’s *t*-test. (**B**) A representative pseudocolor dot plot of an Annexin V and PI staining after 48 h of culture.

CNF1 was originally reported to be cytotoxic but it has subsequently become clear that the toxin, in most cases, promotes the survival of target cells [25]. To asses if CNF1 influenced the viability of moDCs, we analyzed the percentage of cells positive for the apoptotic marker Annexin V and the cell death marker propidium iodide (PI) after 24 and 48 h of culture with 0.1 nM CNF1. As shown in Figure 3, CNF1 did not increase the percentage of Annexin V- and/or PI-positive cells relative to immature DCs cultured without toxin. In contrast, a significant increase in the percentage of Annexin V- and/or PI-positive cells was observed in starved immature moDCs cultured without serum and cytokines (Figure 3). These results indicate that CNF1-mediated maturation of moDCs is not an indirect consequence of compromised cell viability.

**Figure 4:**
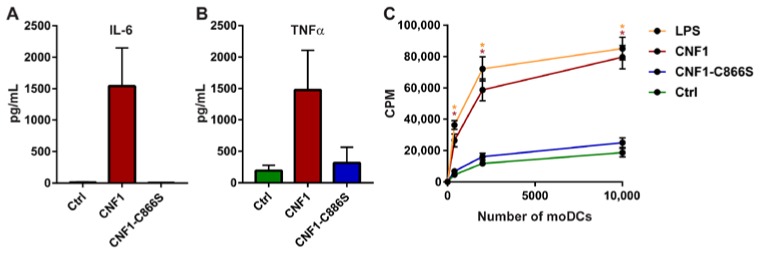
CNF1 induces functional maturation of moDCs. (**A**,**B**) Immature moDCs were cultured for 24 h without toxin (Ctrl), with 0.1 nM CNF1 or with 0.1 nM CNF1-C866S before the concentrations of (**A**) interleukin-6 (IL-6) and (**B**) TNFα in the cell culture supernatants were determined by enzyme-linked immunosorbent assay (ELISA). (**A**,**B**) Shown is the mean cytokine concentration + SEM in cell culture supernatants from 3 independent experiments each using moDCs derived from different donors (*n* = 3). (**C**) Immature moDCs were cultured for 48 h with 0.1 nM CNF1, 0.1 nM CNF1-C866S, 50 ng/mL LPS or without toxin (Ctrl). The moDCs were subsequently washed and different numbers cultured with allogenic naïve CD4+ T cells for 4 days. Then [^3^H]-thymidine was added, and the cells cultured for 24 h further, before determination of [^3^H]-thymidine incorporation. Shown are the mean counts per minute (CPM) from 3 independent experiments. Each independent experiment was performed using moDCs and allogenic naïve CD4+ T cells derived from different donors (total of 6 donors). Error bars represent SEM and * denotes a significant difference (*p* < 0.05) compared to CD4+ T cells cultured with the same number of Ctrl moDCs using a two-way ANOVA with Sidak’s multiple comparisons test.

Based on our findings, it was fundamental to establish if the phenotypic maturation of immature moDCs was accompanied by functional activation and maturation. A hallmark of activated DCs is that they secrete proinflammatory cytokines, such as interleukin-6 (IL-6) and tumor necrosis factor alpha (TNFα) [22]. In keeping with the phenotypic maturation, supernatants from immature moDCs cultured with CNF1 contained highly increased levels of both IL-6 and TNFα when compared to supernatants from immature moDCs cultured without CNF1 or with CNF1-C866S (Figure 4A,B). Another key functional characteristic of mature moDCs is that they are potent in inducing activation and proliferation of naïve CD4+ T cells. Therefore, we investigated the capacity of moDCs that had been cultured with CNF1, CNF1-C866S or as a positive control LPS to induce proliferation of naïve CD4+ T cells isolated from allogenic healthy donors. As shown in Figure 4C, moDCs that had been treated with CNF1 exhibited significantly increased capacity to induce proliferation of allogenic naïve CD4+ T cells relative to moDCs that had been treated with CNF1-C866S or cultured without toxin. Collectively, these findings strongly suggest that CNF1 activity activates immature moDCs to undergo phenotypic and functional maturation.

## 3. Discussion

In recent years, pioneering studies have provided evidence that CNF1 has the capacity to promote inflammation, stimulate host immunity and function as a potent immunoadjuvant [10,11,12,13,14,15,16,17,18]. Nevertheless, the immunostimulatory properties of the toxin are not fully understood and little is known about its direct effects on different immune cell subsets. Here, we show that CNF1 potently triggers activation and maturation of immature human moDCs. CNF1-induced moDC maturation was not associated with increased levels of apoptotic or dead cells, strongly arguing that it was not an indirect effect of compromised cell viability. In contrast to CNF1, the catalytically inactive form of the toxin, CNF1-C866S, failed to trigger moDC maturation. These findings are consistent with prior studies demonstrating that CNF1, but not CNF1-C866S, promotes anti-bacterial immunity and immunization in vivo and supports that the immunostimulatory properties of the toxin are linked to its enzymatic activity [14,15,17,18]. Notably, Diabate et al. found that CNF1 potentiated LPS-induced expression of IL-6 and TNFα from murine monocytes, but that the toxin was unable to trigger secretion of the cytokines in itself [18]. Our results suggest that CNF1 has the capacity to induce secretion of these cytokines from human moDCs independently of LPS, implying that there may be differences in how the inflammatory response to CNF1 is regulated in monocytes and moDCs—or alternatively, in murine and human cells.

The experimental use of primary DCs is limited, due to their severe paucity in peripheral blood, and therefore in vitro-generated moDCs are commonly used as a model [26]. While classical DCs (cDC) are the best characterized, moDCs also represent an important immune cell subset in vivo where they differentiate from circulating monocytes that extravasate from the blood into tissues. Relatively few moDCs are present in tissues and at mucosal surfaces in steady state conditions but their abundance increases drastically during inflammation [21,27]. Once activated, moDCs can remain in the inflamed tissue producing cytokines, killing bacteria and driving local T cell responses or travel to the draining lymph nodes where they may contribute to the activation and differentiation of naïve T cells [21]. It has been proposed that cDCs are central in the initial phase of the inflammatory response, leading to the mobilization of moDCs that are critical for effective clearance of the pathogen in subsequent phases [28]. Although care should be taken when extrapolating findings from moDCs to cDCs, we speculate that CNF1 activity also triggers activation and maturation of cDCs. As *cnf1*-positive *E. coli* strains are generally invasive and capable of infecting diverse host niches, it seems plausible that analogous to monocytes in the blood, DC subsets have the capacity to sense CNF1 activity in tissues and secondary lymphoid organs and in turn promote a protective immune reaction [2,18]. It should, however, be highlighted that CNF1 has also been shown to increase the activity of natural killer cells and to induce secretion of inflammatory factors from endothelial and epithelial cell lines, indicating that several cell types may contribute to CNF1-induced inflammation [29,30,31].

Tissue infiltration of CNF1-expressing bacteria would not only expose DCs to the toxin, but concurrently to a variety of other bacterial factors with the capacity to promote or inhibit DC activation and maturation. Integration of diverse bacteria- and host-induced signals is therefore expected to influence CNF1-driven DC activation in vivo. In line with this idea, Diabate et al. showed that the toxin, α-Hemolysin can protect *E. coli* against CNF1-induced host responses [18]. As α-Hemolysin is often co-expressed with CNF1, this finding interestingly indicates that *E. coli* have evolved strategies to subvert the immunostimulatory properties of CNF1 during infection.

CNF1 is not unique in its ability to tamper with Rho GTPase activity in host cells. Manipulation of Rho GTPase signaling is a conserved strategy utilized by a variety of pathogens to promote invasion and dissemination in the host organism. For example, *Salmonella enterica* serovar Typhimurium expresses the effector protein SopE, which activates Rho GTPases in host cells by serving as a nucleotide-exchange factor [32]. Keestra et al. interestingly demonstrated that SopE-mediated activation of the Rho GTPases Rac1 and Cdc42 triggered the NOD1 signaling pathway in the human embryonic kidney cell line HEK293, eventually leading to activation of the proinflammatory transcription factor NF-κB [33]. Whereas a mutant *sopE*-deficient *S. typhimurium* strain did not elicit remarkable caecal inflammation, *sopE*-proficient *S. typhimurium* induced colitis in wild-type mice, but not in *Nod1*-deficient mice [33]. Indeed, accumulating evidence suggest that excessive Rho GTPase activation provoked by bacterial virulence factors induces formation of a multiprotein complex, involving NOD1 and RIP kinases, which triggers NF-κB activation and cytokine expression. This mechanism is primarily derived from experiments using epithelial cells, but it is feasible that a similar pathway is implicated in CNF1-mediated activation of moDCs. Our findings thus feed into emerging data suggesting that animal cells, similar to effector-triggered immunity in plants, can sense pathogen-induced manipulation of Rho GTPase activity as a danger signal [23,34].

In conclusion, we provide novel evidence showing that CNF1 induces activation and maturation of moDCs. This finding sheds new light on the immunostimulatory properties of CNF1 and supports future prospects for its clinical application as an adjuvant in DC-based vaccines.

## 4. Materials and Methods

### 4.1. Purification of CNF1 and CNF1-C866S

CNF1 was obtained from the 392 ISS *E. coli* strain (kindly provided by Vincenzo Falbo (Istituto Superiore di Sanita, Rome, Italy), and the plasmid coding for CNF1-C866S was a kind gift from Emmanuel Lemichez (Inserm, Nice, France). Both toxins were purified as previously described [8]. The final working concentrations were obtained in Tris-HCl buffer, pH 7.5.

### 4.2. Purification of PBMCs and Generation of Immature moDCs

Peripheral blood mononuclear cells (PBMCs) were isolated by Lymphoprep™ (Axis-Shield, Oslo, Norway) density gradient centrifugation from buffy coats that were obtained from healthy blood donors at the Danish Blood Bank (Department of Clinical Immunology, University Hospital Rigshospitalet, Copenhagen, Denmark). Ethical approval was waived by “Den Videnskabsetiske Komité Københavns og Frederiksberg Kommuner”, which deemed that no results obtained in the experiments were related to the donors, and no samples or cells were stored; samples were identified by date of blood sampling only, and analyzed anonymously. Verbal consent to blood sampling was considered adequate by the Ethics Committee, and was obtained. Monocytes were purified from the PBMCs by negative selection using the Human MACS Monocyte Isolation Kit II from Miltenyi Biotec (Bergisch Gladbach, Germany). The purified monocytes were cultured for 5 days (37 °C and 5% CO_2_) in moDC media (RPMI-1640 AQmedia (Sigma-Aldrich, St. Louis, MO, USA), 100 µg/mL penicillin/streptomycin (Sigma-Aldrich) and 10% endotoxin-low fetal bovine serum (FBS) (ThermoFisher Scientific, Gibco, Waltham, MA, USA)) supplemented with 50 ng/mL recombinant GM-CSF (PeproTech, London, UK) and IL-4 (PeproTech) to generate immature moDCs. The immature moDCs were finally washed and cultured in fresh moDC media supplemented with 50 ng/mL GM-CSF for 24 and/or 48 h in absence (Ctrl) or presence of CNF1, CNF1-C866S, LPS (Sigma-Aldrich), TNFα (PeproTech) or PMB (InvivoGen, San Diego, CA, USA) as indicated prior analysis.

### 4.3. Flow Cytometry

For analysis of maturation markers, cells were resuspended in ice-cold FACS buffer (5% FBS and 0.1% Na-azide in phosphate buffered saline (PBS)) containing diluted mouse serum (Agilent, Santa Clara, CA, USA), stained with BV421-conjugated anti-CD83 (BioLegend, San Diego, CA, USA), APC conjugated anti-CD86 (BioLegend) and PE-conjugated anti-HLA-DR (Leinco, St. Louis, MO, USA) antibodies for 30 min at 4 °C in the dark, washed with FACS buffer and subjected to flow cytometric acquisition. For viability analysis, cells were resuspended in Annexin V Binding Buffer (BioLegend) and stained for 15 min with PE-conjugated Annexin V (BD Biosciences, Franklin Lakes, NJ, USA) at 4 °C in the dark. PI (Biolegend) was subsequently added and the cells subjected to flow cytometric acquisition which was performed at the CFFC (Core Facility for Flow Cytometry, Faculty of Health and Medical Sciences, University of Copenhagen) using a Fortessa 3 flow cytometer (BD Biosciences). The acquired data were analyzed with FlowJo v10 software (Tree Star, Ashland, OR, USA).

### 4.4. CNF1 Depletion

Protein A-agarose macrobeads (Sigma-Aldrich) were coated overnight at 4 °C with an anti-CNF1 antibody (Clone NG8, Hycult Biotech, Plymouth Meeting, PA, USA), an IgG2a isotype control antibody (R&D systems, Minneapolis, MN, USA) or PBS as control. The beads were then washed extensively with PBS and incubated overnight at 4 °C with CNF1 diluted in moDC media. As a negative control, moDC media without CNF1 was incubated in parallel with uncoated control beads. The beads were subsequently pelleted by centrifugation and the supernatants transferred to new tubes before they were incubated with fresh Protein A-agarose macrobeads for 1 h at 4 °C to remove any residual antibody. After removal of the beads, a portion of the supernatants was boiled in reducing sodium dodecyl sulfate sample buffer and analyzed by western blotting, as previously described [35]. The remaining of the supernatants were added to immature moDCs which were analyzed for the expression of CD83 and CD86 after 24 h of culture.

### 4.5. ELISA

The concentrations of IL-6 and TNFα in cell culture supernatants were measured using cytokine-specific human DuoSet Enzyme-Linked Immunosorbent Assay (ELISA) development kits from R&D Systems in accordance with the manufacturer’s instructions.

### 4.6. moDC-Induced Proliferation of Allogenic Naïve CD4+ T Cells

Immature moDCs were cultured in moDC media with 50 ng/mL GM-CSF for 48 h in absence or presence of 0.1 nM CNF1, 0.1 nM CNF1-C866S or 50 ng/mL LPS. The moDCs were subsequently washed and different numbers cultured in X-VIVO 15 media (Lonza, Basel, Switzerland) together with 5 × 10^4^ naïve CD4+ T cells freshly purified from PBMCs from healthy allogenic donors using the Human Naïve CD4+ T cell Isolation Kit II from Miltenyi Biotec. After 4 days of culture, 5 µCi/mL [^3^H]-thymidine (GE Healthcare, Chicago, IL, USA) was added to each sample. The cells were incubated for 24 h further before they were harvested onto UniFilter plates (PerkinElmer, Waltham, MA, USA). Finally, Microscint-20 cocktail (PerkinElmer) was added to the UniFilter plates prior to analysis in a TopCount^®^NXT Microplate Scintillation and Luminescence Counter (PerkinElmer). The [^3^H]-thymidine incorporation was measured as mean counts per minute (CPM) from three replicate cultures.

## Abbreviations

CNF1	Cytotoxic necrotizing factor 1
DCs	Dendritic cells
MAMPs	Microbe-associated molecular patterns
moDCs	Monocyte-derived DCs
PMB	Polymyxin B
LPS	Lipopolysaccharide
cDCs	Classical DCs
PBMCs	Peripheral blood mononuclear cells
IL-6	Interleukin-6
TNFα	Tumor necrosis factor alpha
PBS	Phosphate buffered saline
PI	Propidium iodide
MHC	Major histocompatibility complex
